# Evolutionary Trajectories of Beta-Lactamase CTX-M-1 Cluster Enzymes: Predicting Antibiotic Resistance

**DOI:** 10.1371/journal.ppat.1000735

**Published:** 2010-01-22

**Authors:** Ângela Novais, Iñaki Comas, Fernando Baquero, Rafael Cantón, Teresa M. Coque, Andrés Moya, Fernando González-Candelas, Juan-Carlos Galán

**Affiliations:** 1 Hospital Universitario Ramón y Cajal, IMSALUD, Madrid, Spain; 2 CIBER en Epidemiología y Salud Pública (CIBERESP), Madrid, Spain; 3 Unidad Mixta Genómica y Salud CSISP/UV-Instituto Cavanilles, Valencia, Spain; 4 MRC National Institute for Medical Research, London, United Kingdom; 5 Unidad de Resistencia a Antibióticos y Virulencia Bacteriana asociada al Consejo Superior de Investigaciones Científicas (CSIC), Madrid, Spain; University of Toronto, Canada

## Abstract

Extended-spectrum beta-lactamases (ESBL) constitute a key antibiotic-resistance mechanism affecting Gram-negative bacteria, and also an excellent model for studying evolution in real time. A shift in the epidemiology of ESBLs is being observed, which is characterized by the explosive diversification and increase in frequency of the CTX-M-type β-lactamases in different settings. This provides a unique opportunity for studying a protein evolutionary radiation by the sequential acquisition of specific mutations enhancing protein efficiency and fitness concomitantly. The existence of driver antibiotic molecules favoring protein divergence has been investigated by combining evolutionary analyses and experimental site-specific mutagenesis. Phylogenetic reconstruction with all the CTX-M variants described so far provided a hypothetical evolutionary scenario showing at least three diversification events. CTX-M-3 was likely the enzyme at the origin of the diversification in the CTX-M-1 cluster, which was coincident with positive selection acting on several amino acid positions. Sixty-three CTX-M-3 derivatives containing all combinations of mutations under positively selected positions were constructed, and their phenotypic efficiency was evaluated. The CTX-M-3 diversification process can only be explained in a complex selective landscape with at least two antibiotics (cefotaxime and ceftazidime), indicating the need to invoke mixtures of selective drivers in order to understand the final evolutionary outcome. Under this hypothesis, we found congruent results between the *in silico* and *in vitro* analyses of evolutionary trajectories. Three pathways driving the diversification of CTX-M-3 towards the most complex and efficient variants were identified. Whereas the P167S pathway has limited possibilities of further diversification, the D240G route shows a robust diversification network. In the third route, drift may have played a role in the early stages of CTX-M-3 evolution. Antimicrobial agents should not be considered only as selectors for efficient mechanisms of resistance but also as diversifying agents of the evolutionary trajectories. Different trajectories were identified using a combination of phylogenetic reconstructions and directed mutagenesis analyses, indicating that such an approach might be useful to fulfill the desirable goal of predicting evolutionary trajectories in antimicrobial resistance.

## Author Summary

Antimicrobial resistance in bacterial organisms is a worldwide problem widely discussed from clinical, economical and social points of view. The number of new resistance mechanisms and microorganisms resistant to new drugs is increasing all over the world. The development and spread of antibiotic resistance in bacterial communities represents an excellent model for testing the predictive potential of evolutionary principles at short time-scales. A number of studies have tried to predict the selection of resistant variants when a new drug is commercialized. However, in many cases there is no correlation between *in vitro* predictions and in-field observations. For this reason, it can be suspected that the ability to predict the emergence of new resistant variants might be incomplete unless we know the evolutionary forces acting on the genetic diversification processes. Using the CTX-M β-lactamases as a model, a combination of molecular phylogenetic approaches and experimental site-specific mutagenesis has allowed us to establish evolutionary trajectories. We have demonstrated that two synthetic antibiotics, cefotaxime and ceftazidime, were the selective forces driving the diversification of CTX-M enzymes, but only when both antibiotics were simultaneously present in the environment. We also predict that, if the current selective landscape is not modified, variants carrying the mutation D240G will be more prevalent and diverse in the future.

## Introduction

Antibiotic resistance presently constitutes one of the major factors influencing pathogenesis and the outcome of infections in antibiotic-exposed patients. Resistance is in fact recognized as one of the main problems in public health since it is associated with a high clinical, economic and social impact, and the study of the antibiotic resistant bacteria has been recognized as a high priority intervention area (for additional information see documents “WHO Global strategy for containment of antibiotic resistance” in http://www.who.int/drugresistance/guidance/en/index.html, and “Action Plan to Combat Antimicrobial Resistance” co-chaired by the Centers for Disease Control and Prevention (CDC), the Food and Drug Administration (FDA), and the National Institutes of Health (NIH) in http://www.cdc.gov/DRUGRESISTANCE/actionplan/index.htm). Although strategies for controlling the spread of antibiotic resistance have been suggested and implemented, we have been witnessing an increase in the number and diversity of antibiotic resistance mechanisms. This is what has happened with extended-spectrum β-lactamases (ESBLs), and particularly with the CTX-M-type ESBLs, currently recognized as the most widespread and threatening mechanism of antibiotic resistance, both in clinical and community settings [1]–[3]. Thus, β-lactamases offer one of the best examples of protein diversification and evolution as a mechanism of rapid adaptation of bacterial populations to changing environments [4],[5].

Different studies suggest that CTX-M enzymes derive from chromosomal genes from different *Kluyvera* species, which have subsequently spread among pathogenic and non-pathogenic relevant bacteria [6]–[9]. It has been recently proposed that the relative success of CTX-M enzymes might also be related to the unusually high rate of mobilization of *bla*_CTX-M_ genes from *Kluyvera* species to different genetic platforms, as compared with other class A β-lactamases including those of *bla*_TEM_ or *bla*_SHV_ genes [10]. The successful distribution and prevalence of *bla*_CTX-M_ genes in comparison to classical *bla*_TEM_ or *bla*_SHV_ families in several ecological niches seem to be related to the interplay of different selective forces. Firstly, those derived from the association of *bla*_CTX-M_ genes with particular genetic platforms (insertion sequences, integrons, transposons and plasmids) [1],[11],[12], and/or specific bacterial clones or clonal complexes [13]–[15]. Secondly, the strong selective pressures exerted by the widespread and concomitant use of different β-lactam antibiotics on clinical bacteria, driving the emergence and dispersal of novel co-resistant and more intensely active variants [2],[5],[16],[17].

The adaptive success of particular protein configurations in CTX-M enzymes is known only from the epidemiological point of view, but the underlying reasons driving this phenomenon are still to be understood. To gain insights in this direction, the process of diversification of *bla*_CTX-M_ genes was analyzed from an evolutionary perspective. Previous analyses based on the similarity of the corresponding amino acid sequences have shown that CTX-M ESBLs are grouped into five major clusters (CTX-M-1, -2, -8, -9, and -25) [10],[18]. Since its first description in 1989 [19], more than 80 allelic variants have been recognized, CTX-M-1 and CTX-M-9 being the most diverse clusters (with 31 and 22 variants identified, respectively) (www.lahey.org/studies). The first CTX-M enzymes described displayed a high hydrolytic activity towards cefotaxime (or ceftriaxone), while remaining poorly active in hydrolyzing ceftazidime. Nevertheless, many of the CTX-M variants currently described are able to confer resistance to cefotaxime/ceftriaxone and also to ceftazidime [20]. Here, we hypothesize that exposure to ceftazidime in a selective landscape in which cefotaxime was also present might have constituted the main selective force driving the evolution of CTX-M enzymes. We have focused on the CTX-M-1 cluster since it is the most diversified group and also includes most efficient and ceftazidime-resistant CTX-M variants described to date.

Some previous studies have identified amino acid positions involved in the enhancement and/or modulation of the phenotypic profile of these enzymes [21]–[23], while others have provided important structural insights on the mechanism of action of these broad-spectrum CTX-M enzymes [24],[25]. Nevertheless, we are far from understand the dynamics of different mutational events at the molecular level and the accessible evolutionary pathways that lead to more efficient CTX-M phenotypes, as proposed in other models [26],[27].

The aim of this study was to investigate the different mutational pathways driving the diversification of *bla*_CTX-M-1-like_ genes towards cefotaxime and ceftazidime-resistance phenotypes, and to establish a hypothetical evolutionary scenario for CTX-M-1-cluster enzymes. The study increases our understanding of their evolutionary past and enables us to consider predictive trends in antimicrobial resistance, a need that has been previously claimed [28],[29].

## Results

### Phylogenetic reconstruction and detection of positions under positive selection in the CTX-M-1 cluster

**Figure 1:**
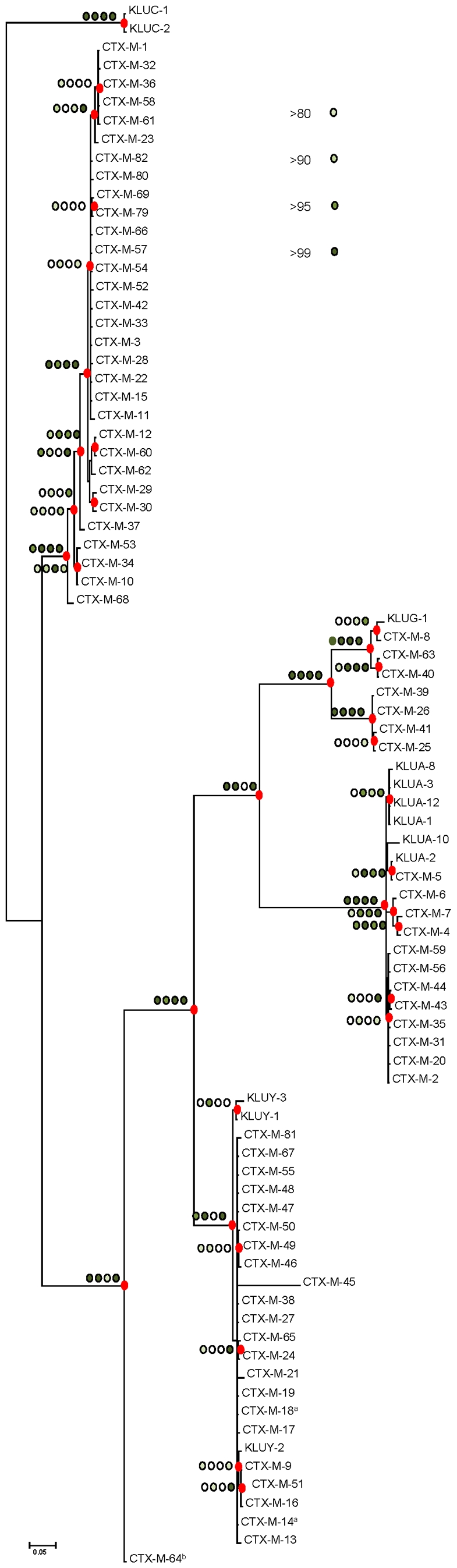
Phylogenetic tree of *bla*_CTX-M_ sequences (n = 73) inferred by Bayesian analysis. Support for relevant nodes in the tree (indicated by a red dot) was estimated by approximate likelihood ratio, bootstrapping (1000 replicates) with maximum-likelihood reconstruction, bootstrapping (1000 replicates) with neighbor-joining reconstruction, and Bayesian posterior probability, color-coded with increasing density of green with the following equivalences: white <0.80, lightest green ≥0.80, light green ≥0.90, medium green ≥0.95 and dark-green ≥0.99. *Kluyvera* spp. *bla* sequences (n = 12) from putative ancestors were included in the analyses and the tree was arbitrarily rooted with *Kluyvera cryocrescens*. The discrepancy detected between this topology and the one generated by Barlow *et al.*, consisting of the position of the *bla*_CTX-M-11_ sequence, might be due to the existence of two different entries in the PubMed corresponding to this *bla* gene: GenBank accession number AY005110 (this study) and AJ310929 [10], differing in six nucleotides and four amino acid changes, probably sufficient to ascribe these variants to different branches. ^a^ *bla*_CTX-M-14_ and *bla*_CTX-M-18_ genes show identical nucleotide sequences; ^b^ *bla*_CTX-M-64_ gene corresponds to a hybrid sequence, probably resulting from a homologous recombination event between *bla*_CTX-M-15_ and *bla*_CTX-M-14_ genes [32].

**Figure 2:**
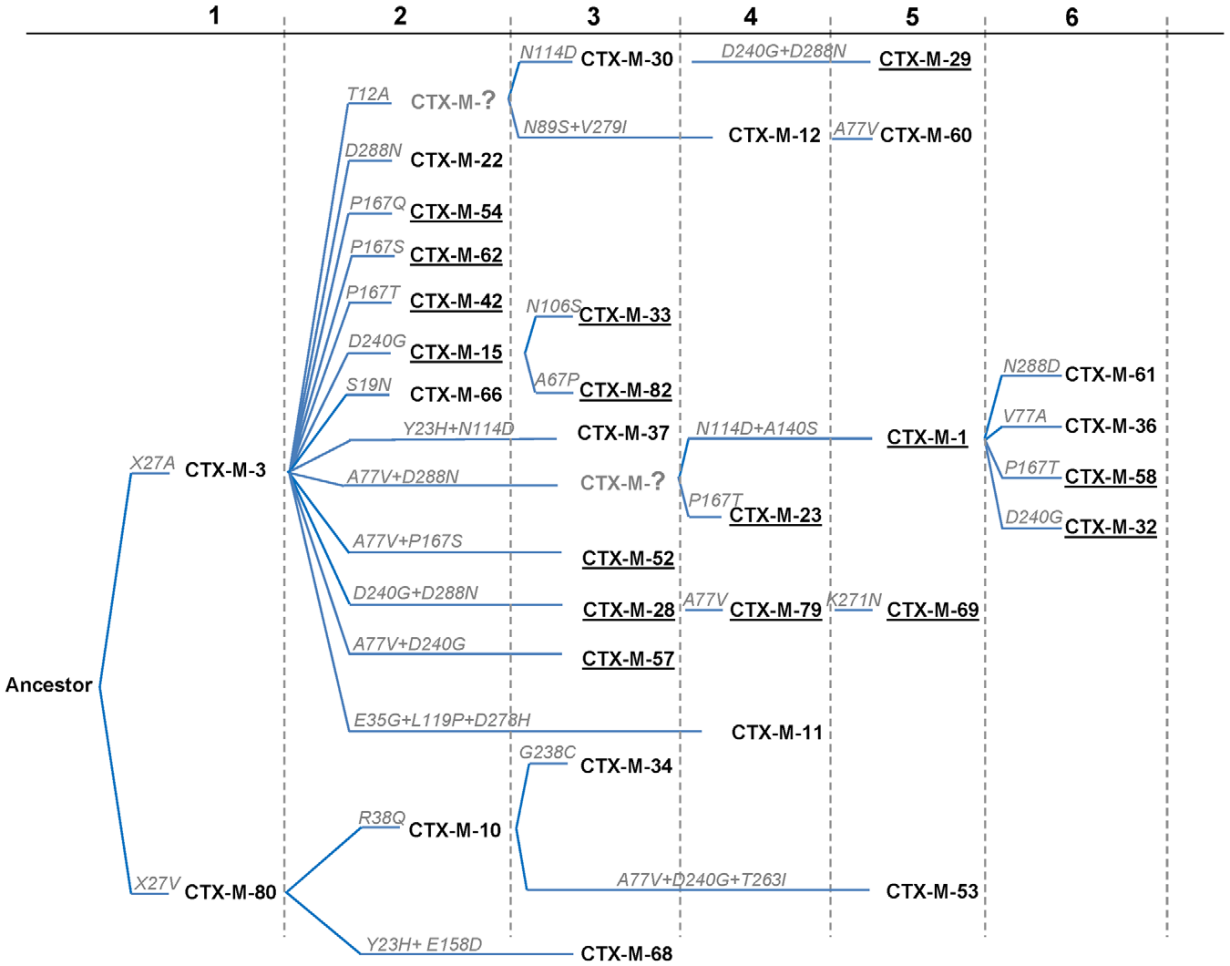
Phylogenetic reconstruction of CTX-M-1 cluster enzymes. Using the MESQUITE 2.0 program, the amino acid sequence of the putative CTX-M-1 ancestor was identified, and subsequently all CTX-M-1 cluster variants were positioned in the generated phylogeny. The amino acid changes acquired in each consecutive step are indicated in each branch. Numbers (n = 1–6) up in the figure represent the cumulative number of amino acid changes present in CTX-M variants with respect to the ancestor sequence. Underlined CTX-Ms represent the variants with activity on both cefotaxime and ceftazidime.

The different phylogenetic tree reconstruction methods used consistently separated the 73 *bla*_CTX-M_ and the 12 *Kluyvera* sequences analyzed into 5 major clusters (Figure 1), confirming previous proposals [10],[18]. The most closely related sequences to the CTX-M-1 cluster, *bla*_KLUC-1_ and *bla*_KLUC-2_ from *K. cryocrescens* [6],[10],[18], were consistently located outside this cluster and the distance from their hypothetical common ancestor sequence suggests that the real ancestor of *bla*_CTX-M-1-like_ genes might still be unidentified. However, based on this phylogenetic tree, a putative ancestral amino acid sequence of the CTX-M-1 cluster was obtained and sequences in the successive nodes derived from this hypothetical ancestor could also be inferred (Figure 2). According to this representation, CTX-M-3 and CTX-M-80 β-lactamases were located in the deepest nodes of the phylogenetic tree. This hypothetical evolutionary scenario served as reference for developing our experimental approach.

The phylogenetic analysis showed a successful diversification of the CTX-M-3 β-lactamase lineage, with at least 25 CTX-M-3 β-lactamase derivatives. Among these, 64% (16/25) confer resistance to both cefotaxime and ceftazidime (Figure 2), suggesting that the diversification process might have been of an adaptive nature, providing advantages to the harboring strains in a complex clinical-therapeutical environment. If an adaptive process underlies the fast diversification of CTX-M-3, one or several amino acid positions of this protein might be under positive selection (ω = dN/dS >1). So, the analysis of sequences associated with the CTX-M-1 cluster (Figure 1) would indicate positions with a ω higher than 1, suggesting a fast adaptation of the organisms producing CTX-M-3-derived enzymes to novel selective challenges. Our analysis showed that the amino acid positions under positive selection were 77, 114, 167, 240 and 288. On the other hand, when we analyzed the relative rates of non-synonymous to synonymous substitutions at those sites for the remaining sequences of the phylogenetic tree for all CTX-M cluster enzymes, ω was lower than 1, thus indicating that purifying selection was the driving force in these lineages. This result suggests a slower adaptive evolutionary rate at the beginning of the diversification process as compared to the faster amino acid replacement rate observed for the most recent CTX-M-3-derivative sequences.

### Diversification in the CTX-M-1 cluster: Experimental reconstruction

In order to assess the influence of the positions predicted to be under positive selection in CTX-M-1 cluster enzymes on the increasing bacterial fitness in environments contaminated with ceftazidime, specific mutations in each predicted site (A77V, N114D, P167S, D240G and D288N) were introduced by site-directed mutagenesis into the CTX-M-3 background. A phenotypic test (based on the variation of the resistance patterns observed for different β-lactam antibiotics, especially cefotaxime and ceftazidime) was carried out. Although the change A140S was not predicted to be under positive selection, we decided to include it in this analysis as it appears together with putative positively selected changes in CTX-M-1, which is included in the most diversified trajectory (from CTX-M-3) (Figure 2). Therefore, sixty-three artificial CTX-M-3 variants containing all combinations of these six changes were constructed (Table 1, suppl. Table S1). Experimental evolution studies of strains harboring CTX-M-3 in the presence of increasing ceftazidime concentrations have shown that mutations in positions 167 (P167S/T, both providing undistinguishable phenotypes) and 240 (D240G, the unique amino acid change occurring in this position) are the first ones to be selected [21],[23], and constitute the most efficient variants in hydrolyzing ceftazidime [1],[5],[16]. This indicates that the P167S/T and D240G changes might represent the early steps of two independent pathways for the diversification of CTX-M-3 enzymes. Despite the simultaneous presence of these mutations has been shown to produce deleterious effects [23], we cannot conclude that the pleiotropic antagonism observed between P167S and D240G mutations would take place in all mutational combinations and, in consequence, we tested them in all possible genetic backgrounds. All of them showed decreased hydrolytic activity against cefotaxime and/or ceftazidime in comparison with their predecessors carrying only P167S or D240G changes (suppl. Table S1).

**Table 1:** Amino acid changes and β-lactam MIC values associated with CTX-M-3 derivatives constructed in this study.

Mutant^c^	GenBank Acc. N°^d^	CTX-M variant^e^	Amino acid changes^a^						MIC values (µg/ml) of different β-lactam antibiotics tested^b^						
			A77V	N114D	A140S	P167S	D240G	D288N	CTX	CAZ	FEP	CXM	CEF	FXT	AMC
000000	Y10278	CTX-M-3							256	1	32	>256	>256	2	8
100000	GU125712		+						128	1	1.5	>256	>256	0.5	6
010000	GU125670	CTX-M-30^f^		+					32	1	8	>256	>256	0.5	8
001000	GU125671				+				32	1	4	>256	>256	1.5	8
000100	GU125668EF219134	CTX-M-62				+			1	32	0.5	128	192	2	8
000010	AY044436	CTX-M-15					+		128	2	1	>256	256	2	4
000001	GU125665AY080894	CTX-M-22						+	24	1	2	>256	>256	1.5	8
110000	GU125683		+	+					>256	2	6	>256	>256	1.5	8
101000	GU125696		+		+				256	1.5	2	>256	>256	1	6
100100	GU125667DQ223685	CTX-M-52	+			+			4	32	0.25	64	>256	2	4
100010	GU125662DQ810789	CTX-M-57	+				+		>256	6	1	>256	>256	1.5	4
100001	GU125680		+					+	64	1.5	3	>256	>256	2	8
011000	GU125697			+	+				6	1.5	4	>256	>256	1.5	6
010100	GU125672			+		+			8	>256	4	>256	>256	1.5	16
010010	GU125677			+			+		256	12	3	>256	>256	1.5	4
010001	GU125675			+				+	24	1	8	>256	>256	2	6
001100	GU125669				+	+			1	16	0.5	64	192	3	8
001010	GU125658				+		+		64	8	1.5	>256	>256	1	4
001001	GU125661				+			+	8	1	8	>256	>256	1	8
000101	GU125676					+		+	0.5	8	0.094	256	192	1	4
000011	GU125664AJ549244	CTX-M-28					+	+	128	4	1.5	>256	>256	1	3
111000	GU125663EF219142	CTX-M-61	+	+	+				16	1.5	0.38	>256	>256	1	4
110100	GU125673		+	+		+			8	64	0.5	256	>256	2	4
110010	GU125689		+	+			+		>256	8	4	>256	>256	1.5	3
110001	GU125693		+	+				+	128	2	3	>256	>256	3	8
101100	GU125674		+		+	+			4	48	0.25	64	>256	2	4
101010	GU125690		+		+		+		256	8	1.5	>256	>256	1	3
101001	GU125703		+		+			+	32	1.5	2	>256	>256	1	8
100101	GU125660	CTX-M-23^f^	+			+		+	2	48	0.38	>256	128	1	4
100011	GU125666EF426798	CTX-M-79	+				+	+	256	6	>256	>256	>256	1.5	4
011100	GU125659			+	+	+	+		0.5	12	0.5	>256	128	1.5	4
011010	GU125684			+	+		+		256	12	3	>256	>256	1	4
011001	GU125657AB177384	CTX-M-36		+	+			+	12	1.5	1	>256	>256	3	4
010101	GU125682			+		+		+	1	16	0.25	>256	128	2	4
010011	GU125706	CTX-M-29^f^		+			+	+	12	4	0.5	>256	>256	1.5	4
001101	GU125692				+	+		+	2	32	0.25	>256	>256	2	8
001011	GU125686				+		+	+	6	2	0.38	>256	>256	1.5	2
111100	GU125701		+	+	+	+			3	96	0.38	256	>256	1.5	4
111010	GU125704		+	+	+		+		>256	12	4	>256	>256	1	4
111001	X92506	CTX-M-1	+	+	+			+	>256	4	128	>256	>256	1.5	8
110101	GU125698		+	+		+		+	2	96	0.5	>256	128	2	4
110011	GU125687		+	+			+	+	>256	24	16	>256	>256	3	4
101101	GU125699		+		+	+		+	1.5	32	0.25	>256	128	1	4
101011	GU125702		+		+		+	+	256	4	2	>256	>256	0.75	3
011101	GU125679			+	+	+		+	0.2	3	0.125	>256	96	2	4
011011	GU125707			+	+		+	+	12	1.5	0.25	>256	>256	1.5	4
111101	GU125713	CTX-M-58^f^	+	+	+	+		+	12	256	4	>256	>256	2	16
111011	AJ557142	CTX-M-32	+	+	+		+	+	256	8	6	>256	>256	2	4

a Amino acid changes corresponding to non-synonymous substitutions introduced by site-directed mutagenesis in CTX-M-3 in order to obtain the corresponding CTX-M allele, “+” indicates presence.

b MIC values as determined by E-test to the following β-lactam antibiotics: CTX, cefotaxime; CAZ, ceftazidime; FEP, cefepime; CXM, cefuroxime; CEF: cephalotin; FXT: cefoxitine; AMC: amoxicillin-clavulanic acid (2∶1).

c The binary code indicates the content of mutations in each CTX-M variant in this order: A77V, N114D, A140S, P167S, D240G and D288N.

d GenBank accession numbers of *bla*_CTX-M_ alleles constructed by site-directed mutagenesis in this work and previously described.

e CTX-M-3 derivatives corresponding to natural variants are shown.

f Variants closely related to others described in nature (differing in only one mutation or presenting a different amino acid change).

**Figure 3:**
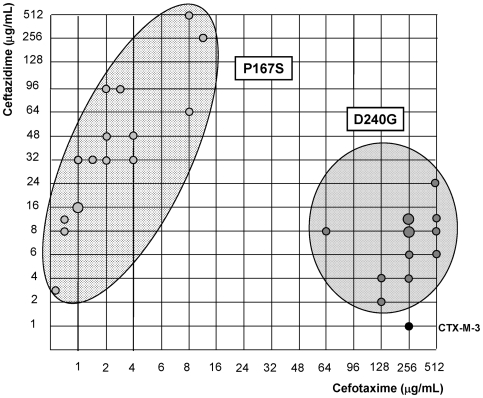
Schematic representation of most CTX-M-3 derivative mutants containing P167S or D240G amino acid changes. Light grey dots represent P167S-carrying mutants, while dark grey dots represent D240G-containing mutants. The size of the dots is directly proportional to the number of mutants represented in each position (1–2). Their corresponding cefotaxime and ceftazidime MIC values (µg/ml) are shown in the “x” and “y” axis respectively.

Among all the antibiotics tested, we only observed significant changes in the resistance levels to cefotaxime and ceftazidime, whereas the minimum inhibitory concentration (MIC) values of other β-lactams were only modestly affected (Table 1). CTX-M-3 derivatives constructed by site-directed mutagenesis were thus ordered towards the most complex (those containing a combination of all mutations analyzed) and/or successful mutants (increased MIC values to ceftazidime and/or cefotaxime) leading to the identification of different presumptive evolutionary trajectories. The combination of P167S with any other mutation predicted to be under positive selection consistently produced drastic changes in cefotaxime and/or ceftazidime MICs. On the contrary, D240G in combination with the same mutations produced mild changes in the activity of the derived enzymes on the same antibiotics (Figure 3).

### Diversification of CTX-M-1 cluster along the P167 pathway

**Figure 4:**
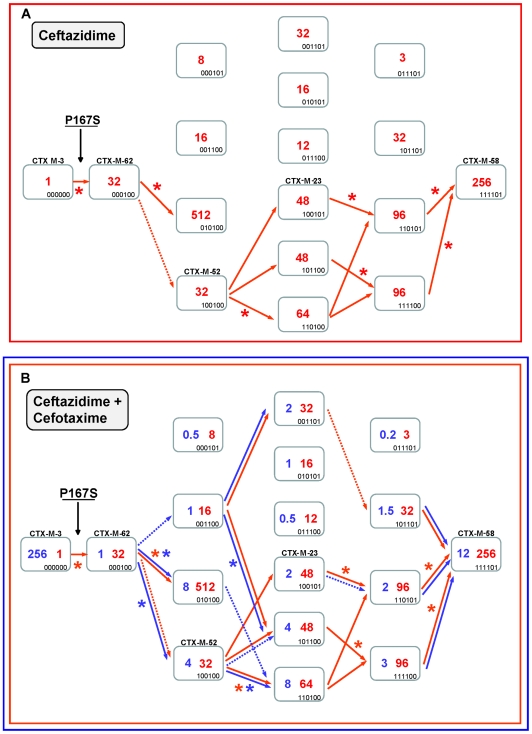
Mutational pathways showing the diversification of CTX-M-3 towards the most evolved variants, determined by step-by-step site-specific mutagenesis: the P167 pathway. Different CTX-M-3 evolutionary scenarios are shown depending on the acquisition of P167S (Figure 4) or D240G (Figure 5) mutations in the first step or in the absence of these changes (Figure 7). Each rectangle represents a particular CTX-M-3 derivative obtained by introduction of mutations predicted to be under positive selection (A77V, N114D, P167S, D240G and D288N) and A140S in cumulative steps towards the most complex mutant (containing all mutations analyzed in each pathway). The binary code indicates the content of mutations in each CTX-M variant in this order: A77V, N114D, A140S, P167S, D240G and D288N. Variants identical or closely related with enzymes already detected in nature appear with the corresponding CTX-M designation. Numbers inside the rectangles indicate MIC values (µg/ml) of ceftazidime (A) or both cefotaxime and ceftazidime (B) observed for MI1443 *E. coli* cells carrying a recombinant plasmid containing each corresponding mutant. Possible trajectories identified are shown with arrows. Filled arrows indicate evolutionary steps predicted to be favored by selection, whereas dotted arrows represent steps associated with neutral events and thus fixed by drift. Deleterious steps (associated with negative changes in fitness) are not shown. Asterisks indicate significant (using Mann-Whitney tests) increases in MIC between the corresponding mutants.

The diversification of CTX-M-3 enzymes was evaluated considering the acquisition of the P167S change in the first step, where ceftazidime seems to be the main driver (from CTX-M-3 to CTX-M-62) (Figure 4A). This change produces a drastic loss of enzymatic activity against cefotaxime (from 256 µg/ml to 1 µg/ml), suggesting that this variant would only be favored by selection in environments with high exposure to ceftazidime. Among the subsequent fifteen possible CTX-M-62 derivatives (Figure 4), seven showed a higher efficiency against ceftazidime. In addition, the analysis of the deduced diversification trajectories from CTX-M-62 suggests that only a few mutational pathways are expected to occur by adaptive evolution. The most complex enzyme included in this pathway is the mutant CTX-M-3+P167S+A77V+N114D+A140S+D288N (closely related to the natural variant CTX-M-58), which confers higher MIC values to both ceftazidime and cefotaxime than CTX-M-62 (256 µg/ml *versus* 32 µg/ml, and 12 µg/ml *versus* 1 µg/ml, respectively).

Ceftazidime could also have played a key role in the ensuing diversification events since four routes from CTX-M-62 to CTX-M-58 are possible (Figure 4A). All of them involve a common step, the acquisition of the change A77V (CTX-M-52, see Figure 4A), which results in an unchanged ceftazidime MIC value, suggesting that its emergence was most likely due to genetic drift in finite populations and a ceftazidime dominant selective landscape. This double mutant has already been detected both in nature and in laboratory conditions [23]. In the next step, the fixation of N114D, A140S or D288N in this background (CTX-M-52) resulted in increased ceftazidime hydrolytic effects (from 32 to 48–64 µg/ml). In a similar way, an improved ceftazidime phenotype was observed in the subsequent steps towards the most evolved and hydrolytically efficient variant (CTX-M-58, 256 µg/ml), indicating that almost all trajectories are possible. Indeed, one of them includes mutant P167S+A77V+D288N, a derivative previously described in nature (CTX-M-23). In summary, different trajectories under ceftazidime selective pressure might have occurred, all of them including CTX-M-52 (P167S+A77V), which constitutes the mutant with a low probability of being favored by selection. However, the introduction of cefotaxime in the previous selective landscape could explain its selection, since it shows an increased activity against cefotaxime (from 1 to 4 µg/ml) partially restoring the loss of cefotaxime activity observed in the first step (CTX-M-3 to CTX-M-62, from 256 µg/ml to 1 µg/ml). This suggests that cefotaxime exposure could also have played a directional role in the early stages of the diversification process. However, the evolution from CTX-M-62 to CTX-M-58 in a hypothetical environment containing only cefotaxime is difficult to explain under the hypothesis of antibiotic selection (indeed, none of the hypothetical intermediates has been found in nature).

Therefore, only the simultaneous presence of both antibiotics could explain in a satisfactory way the diversification process leading from CTX-M-3 to CTX-M-58, identifying eight possible routes (Figure 4B). According to the statistical analysis, the most probable routes would be P167S+A77V+N114D+A140S (or D288N)+D288N (or A140S) (Figure 4B). Early in the diversification process, the double mutant CTX-M-3+P167S+N114D yielded ceftazidime and cefotaxime MIC values similar to those observed for CTX-M-58 and would be selectable according to our model. Surprisingly, this putative CTX-M variant has not yet been detected in nature. It is noteworthy that although the presence of P167S confers the highest ceftazidime MICs, only few of its derivatives might result in beneficial variations in a mixed ceftazidime-cefotaxime/ceftriaxone selective landscape. If such selective framework is maintained in the clinical environment, it would suggest a limited possibility of evolution and emergence of new variants in β-lactamases containing P167S/T mutations in the future.

### Diversification of CTX-M-1 cluster along the D240 pathway

**Figure 5:**
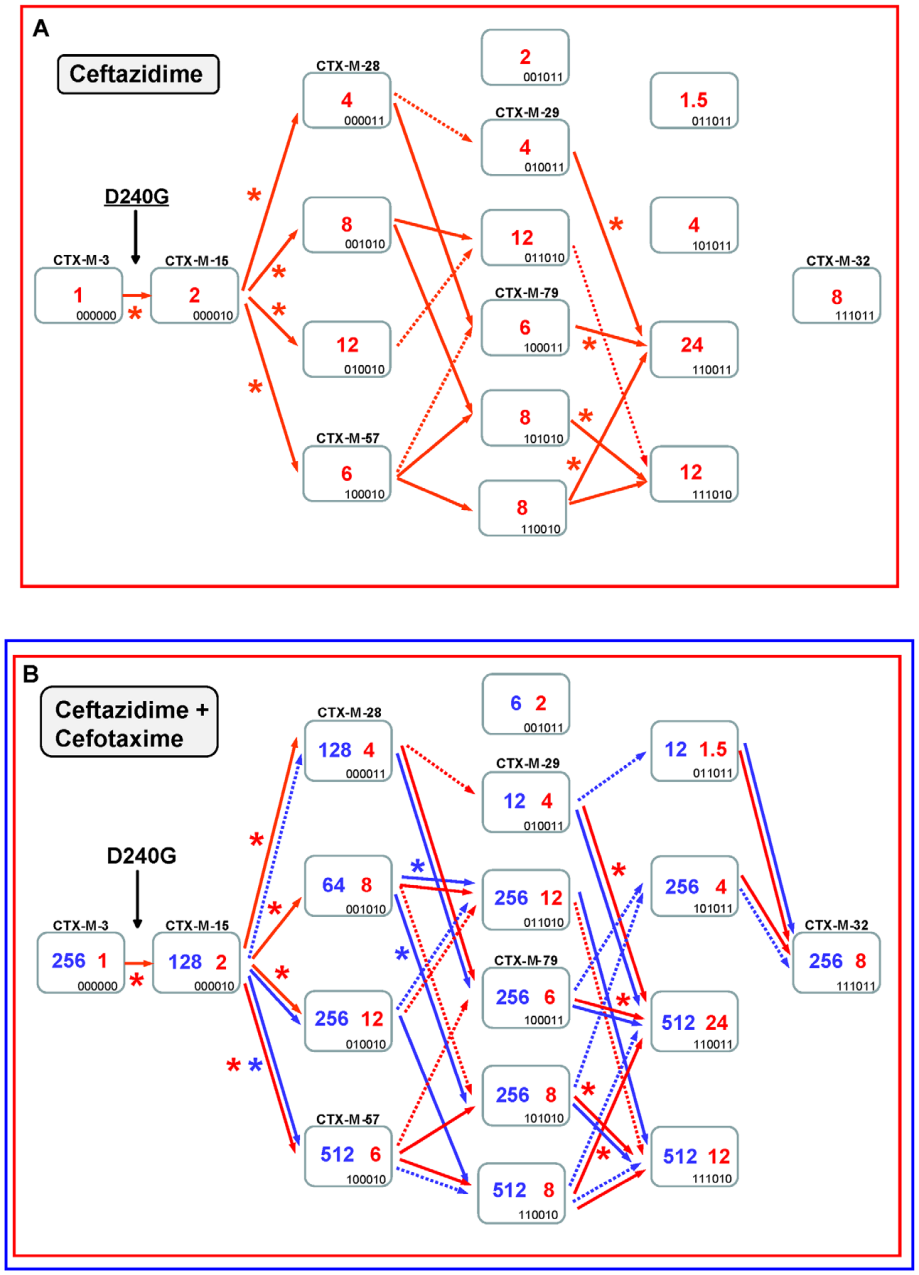
Mutational pathways showing the diversification of CTX-M-3 towards the most evolved variants, determined by step-by-step site-specific mutagenesis: the D240 pathway. See legend for Figure 4.

The diversification of CTX-M-3 enzymes was also analyzed considering the initial acquisition of the D240G change. In the first step, the acquisition of D240G by the CTX-M-3 enzyme (corresponding to CTX-M-15) resulted in a small increase in the efficiency in hydrolyzing ceftazidime (from 1 to 2 µg/ml). The diversification of CTX-M-3 through this route might have occurred within a more complex scenario than that proposed above for the P167 pathway since many (n = 13) variants showed higher (although modest) efficiency in hydrolyzing ceftazidime than CTX-M-15 (Figure 5). Indeed, if ceftazidime were the main selector agent driving the diversification through the D240 path, at least nine different mutational trajectories could be identified towards the most efficient and/or complex variants (Figure 5A).

In the second mutagenesis step, the introduction of the single amino acid changes A77V, N114D, A140S and D288N in CTX-M-15 (CTX-M-3+D240G) yielded variants with higher hydrolytic activity on ceftazidime (from 2 to 4–12 µg/ml) in each case, which suggests that any of these mutants might have been favored by selection. Among these, two have already been found in nature: CTX-M-15+A77V (CTX-M-57) and CTX-M-15+D288N (CTX-M-28), whereas the most efficient mutant (CTX-M-15+N114D) has not yet been described (Figure 5A). After a third mutagenesis step, most resulting combinations could be favored in a ceftazidime selective environment. Among the selectable combinations, we can find the CTX-M-28+N114D variant (CTX-M-29) and the mutant CTX-M-57+D288N, whose emergence we previously predicted [30] and has been subsequently found in nature (CTX-M-79) [31]. In the fourth directed mutagenesis step, two out of the four possible derivatives could be favored by ceftazidime. One of them, the mutant D240G+A77V+N114D+D288N, eventually derived from CTX-M-29, CTX-M-79, or other yet undescribed intermediary, carries almost all positions under positive selection (except P167S) and constitutes the most efficient variant included in this pathway (ceftazidime MIC value = 24 µg/ml), suggesting that it could be easily favored by selection.

According to this model, CTX-M-32 (the most complex variant of this pathway) would not be favored in environments where ceftazidime is the only selective agent. Nevertheless, and in a similar way to that proposed for the P167 pathway, the introduction of cefotaxime in the selective landscape would explain more easily the emergence of this variant (Figure 5B). In this hypothetical dual ceftazidime-cefotaxime scenario, the emergence of the other two intermediates of CTX-M-32 (from CTX-M-29, CTX-M-79 or from the mutant D240G+A77V+A140S) could be favored by selection. Positive selection of CTX-M-32 would occur in the last step after acquisition of mutation N114D or A77V under exposure to ceftazidime and/or cefotaxime (Figure 5B). Hence, different possible routes were identified from CTX-M-3 to CTX-M-32 by sequential acquisition of D240G, D288N, N114D, A140S and finally A77V, or D240G, A77V, A140S (or D288N), D288N (or A140S) and finally N114D. These pathways include four already described variants (CTX-M-28, CTX-M-29, CTX-M-57 and CTX-M-79), supporting that this scenario might have occurred in natural contexts. On the other hand, the most significant trajectories supported by the statistical analysis involved i) the sequential acquisition of D240G, A140S, A77V and N114D; and ii) D240G, D288N, A77V and N114D, the latter including three already detected variants (CTX-M-15, CTX-M-28, CTX-M-79).

It is of note that using this model of evolutionary trajectories we were able to predict many D240G-containing CTX-M-3 derivatives previously detected in nature, which prompts us to suggest that more D240G-carrying variants might be identified in the future. In comparison with the P167 path, the D240 evolutionary pathway is a more plastic route but with limited phenotypic effects, where the acquisition of different mutations and the build-up of different mutational combinations have similar phenotypic outcomes (Figure 3).

### A complex network of diversification: from CTX-M-3 to CTX-M-1

**Figure 6:**
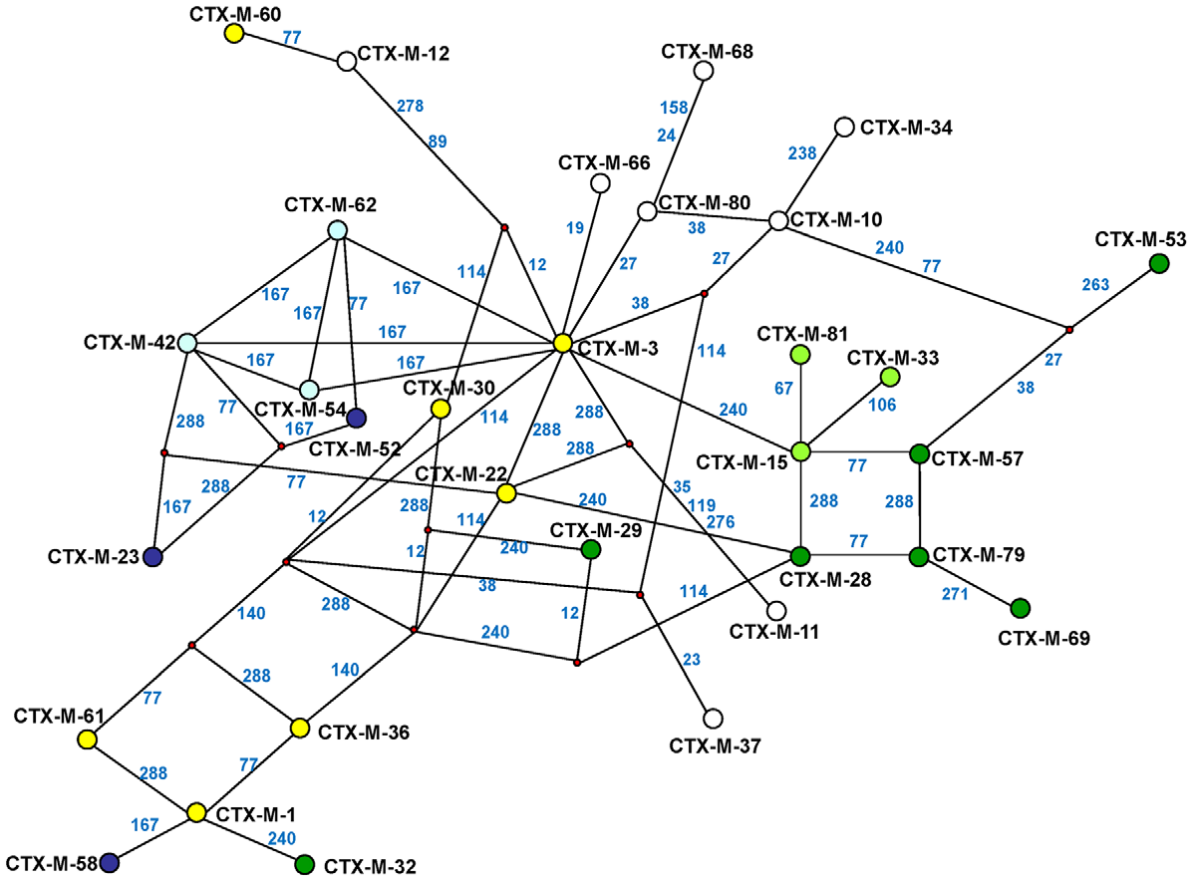
Median-joining network inferred for CTX-M-1 group amino acid sequences. Numbers next to the segments connecting nodes denote the amino acid position inferred to have changed between the two connected alleles. Large dots represent CTX-M variants detected in natural contexts, which differ in the presence of mutations in positions evolving under positive selection (A77V, N114D, P167S, D240G, D288N) as follows: blue dots represent variants containing the P167S mutation alone (light blue) or associated with others (dark blue); green dots represent alleles containing the D240G change alone (light green) or associated with others (dark green); yellow dots represent variants containing mutations A77V, N114D and/or D288N; white dots represent variants containing mutations in other positions presumptively not subjected to positive selection. Alleles whose existence is postulated by the analysis but which have not yet been identified are represented by small red dots in the corresponding nodes.

The experimental reconstruction based on directed mutagenesis of the P167 and D240 pathways suggests that it is possible to attain the most complex and successful variants via several alternative routes. While not all of them have necessarily occurred, some are interconnected, raising difficulties in reconstructing a single evolutionary history for *bla*_CTX-M-1-like_ genes. A possible alternative reconstruction of the phylogenetic relationships among CTX-M-1 cluster enzymes was obtained by median-joining network analysis (Figure 6). Although such an analysis does not allow inferring the ancestrality of the whole set of alleles, those that occupy central positions are considered to represent the most likely ancestors of each group. Accordingly, CTX-M-3 seems to be next to the basal line (oldest variant) since it is at one amino acid change distance from seven known alleles and four yet-unknown (intermediate) variants, and its average distance to the remaining alleles in this group is the lowest (1–5 changes). This observation is congruent with the maximum-likelihood tree shown in Figure 2 and with the results presented by Barlow *et al.* [10] (see above). The network is characterized by a high level of homoplasy, involving only a few amino acid positions such as 167, 240 and 288, which indicates that a minimum number of amino acid changes explains the derivation of any allele from its ancestors in the group.

**Figure 7:**
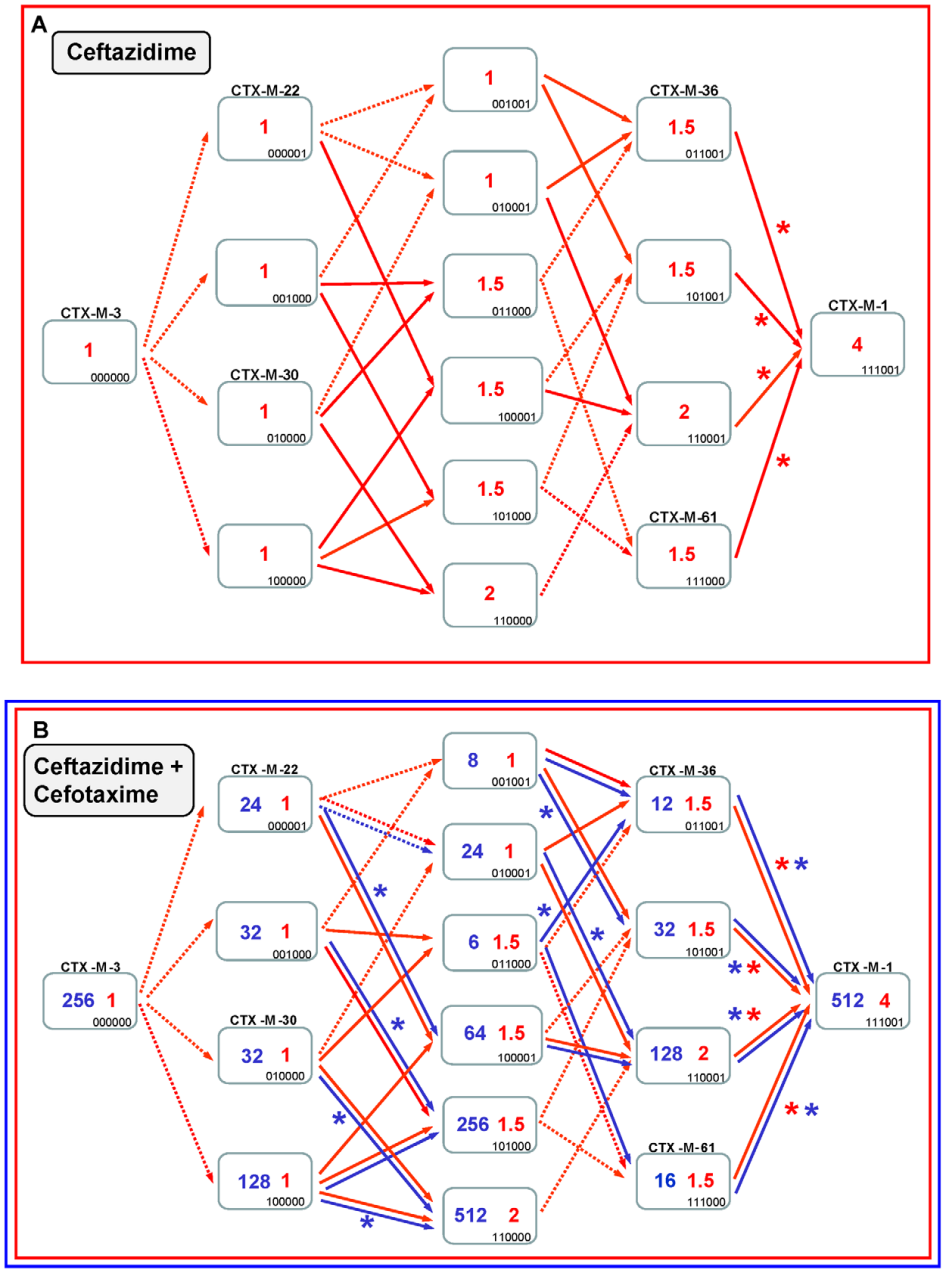
Diversification of CTX-M-3 towards CTX-M-1 in the absence of P167S and D240G. See legend for Figure 4.

Independence between the two main previously described pathways P167 and D240 may be suspected from the analysis of Figure 6, as the only common nodes among them consist of CTX-M-3 (oldest variant) and CTX-M-22 (CTX-M-3+D288N). The median-joining network analysis could suggest a third possible diversification pathway, leading to the evolution from CTX-M-3 towards CTX-M-1 from which CTX-M-58 or CTX-M-32 could easily emerge through the acquisition of P167T or D240G mutations, respectively, in the last step. CTX-M-1 differs from its putative ancestor (CTX-M-3) by four amino acid changes (A77V, N114D, A140S and D288N), three of which (A77V, N114D and D288N) were previously associated with positive selection processes through ceftazidime and cefotaxime exposure, suggesting that similar selective forces have contributed to the diversification from CTX-M-3 to CTX-M-1. Since CTX-M-1 yielded a four-fold increase (from 1 to 4 µg/ml) in the MIC value for ceftazidime, the sequential acquisition of mutational changes from CTX-M-3 leading to CTX-M-1 was analyzed experimentally and the possible hypothetical evolutionary scenarios were considered (Figure 7).

**Figure 8:**
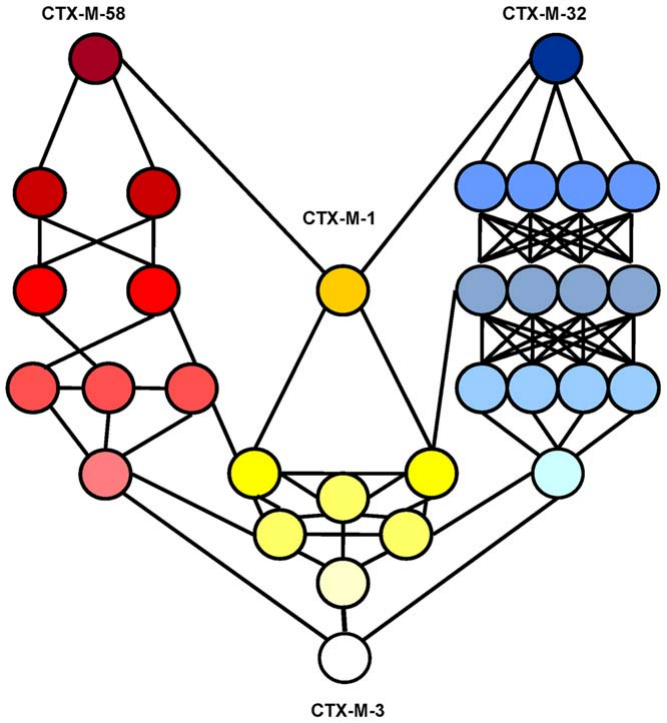
Interconnectedness of three CTX-M-3 diversification routes. Circles represent CTX-M-3 mutant derivatives associated with the different evolutionary routes towards the most evolved variants: i) P167 pathway (blue), ii) D240 pathway (green) and iii) evolution towards CTX-M-1 (yellow). The color gradient represents the direction of evolution.

If ceftazidime were the only selective driver, the introduction in the first step of each single mutation in the *bla*_CTX-M-3_ background by means of site-directed mutagenesis did not yield any variant with increased activity on ceftazidime (Figure 7A). In the second and consecutive steps, all possible intermediates including also CTX-M-1 would arise in the population. Although some of these intermediates might emerge through positive selection (favored by small increases in ceftazidime MIC values), all hypothetical trajectories include at least one intermediate that can only be explained by drift (e.g. the known variants CTX-M-22, CTX-M-30 or CTX-M-61). If we consider a heterogeneous ceftazidime-cefotaxime environment, as proposed in the two main P167 and D240 pathways (see above), only 6 trajectories in the evolution from CTX-M-3 to CTX-M-1 involve steps favored by selection, with the sole exception of the first step, reducing the probability of these trajectories to have occurred in a selective environment (Figure 7B). According to the median-joining network reconstruction of the enzymes in the CTX-M-1 cluster already detected in nature (Figure 6), one of these six trajectories might have occurred. This possible route involves the neutral acquisition of N114D in the first step. In a second step, the introduction of the A140S mutation would result in a slight increase in the ceftazidime MIC value (from 1 to 1.5 µg/ml), while the cefotaxime resistance level would decrease (from 32 to 6 µg/ml). In the next step, the introduction of A77V or D288N would yield CTX-M-61 and CTX-M-36 variants respectively, partially restoring cefotaxime-hydrolyzing activity (from 6 to 12–16 µg/ml). Finally, the introduction in the last step of D288N or A77V, respectively, would generate CTX-M-1 (Figure 6, Figure 7B). From CTX-M-1, the highly efficient enzymes CTX-M-32 (CTX-M-1+D240G) and CTX-M-58 (CTX-M-1+P167T) could easily emerge. In addition, the introduction of P167S or D240G could also have occurred at intermediate stages, as inferred by both the site-specific mutagenesis approach and the median-joining network analysis. For instance, the emergence of CTX-M-23 or CTX-M-28 β-lactamases (included in the P167S and D240G, respectively), could also have occurred in CTX-M-3 by the sequential acquisition of D288N+A77V+P167S or D288N+D240G, respectively (Figure 2), indicating the possibility of connections between this third path and the two main routes (Figure 8).

## Discussion

In this project, the possible existence of selective antibiotic drivers leading to the diversification of CTX-M-3 derivative enzymes has been studied. Our data indicate that the exposure of bacterial populations to synchronic or diachronic challenges with ceftazidime *and* cefotaxime seems to have played a critical role in the explosive diversification of CTX-M variants, and we suggest that the most evolved enzymes are those with high-level, balanced resistance to both drugs. Although we have not observed significant changes in the MIC values of other clinically used β-lactam antibiotics tested (Table 1), we cannot totally exclude the possible contribution of other antibiotics in the diversification process (e.g. cephalosporins of veterinary use) [17]. Similarly, alternative explanations cannot be discarded, including diversifying selection events, such as recombination [32], or alternative selective processes, such as possible physiological effects of some mutations in specific CTX-M variants in the absence of antibiotic exposure [33].

A mutational landscape model [34] similar to the one proposed to explore the step-by-step molecular evolution of TEM β-lactamases [27] was applied here to understand the evolution of CTX-M. The basic hypotheses were: i) a higher fitness is provided by mutations increasing both cefotaxime *and* ceftazidime MICs in environments contaminated by these antibiotics; and ii) consequently, the direction of the evolution of CTX-M-3-derived variants should be towards those yielding higher joint MICs, such as CTX-M-32. The assumption that relates higher fitness with increased MICs is based on two notions: a) only organisms able to resist high antibiotic concentrations will survive in environments in which these concentrations are frequently present (such as urine); b) organisms able to resist high β-lactam concentrations should be selected over those with lower resistance levels in antibiotic gradients that are formed on human surfaces and tissues [35]. Considering the larger extension of the areas with low antibiotic concentrations, high-resistance levels ensure higher fitness in wider spaces. Increased fitness derives from this “larger selective compartment” [36],[37].

CTX-M mutants containing the change P167S seem to be the most successful in generating highly effective variants acting on ceftazidime in a few steps (Figure 3). However, out of the thirty-one possible mutational combinations from CTX-M-62 (CTX-M-3+P167S) constructed in this work, only fifteen yielded an increased efficiency at hydrolyzing ceftazidime and/or cefotaxime, and only three of them have been detected in nature (Figure 4, suppl. Table S1). The analysis carried out in this study indicates that only a few mutational routes towards the highest fitness peak variants are reachable by Darwinian selection, and that many of the remaining trajectories have very low probabilities of realization, as it was shown with TEM-enzymes [27],[38]. Such constraints in this evolutionary network indicate that once optimum or near-optimum variants are readily selected (as the CTX-M-3 variant carrying P167S+N114D changes), novel mutants emerging on the peak will precipitously decrease their efficiency in a ceftazidime-cefotaxime environment, which can be illustrated as the existence of single high fitness peak surrounded by steep cliffs [26].

On the contrary, mutants included in the D240 pathway showed modest effects on fitness, which suggests that many alternative trajectories with common intermediate genotypes might produce a similar phenotype (Figures 3 and 5). Therefore, these trajectories are connected and their connectedness implies (results in) some degree of robustness in the system [39]. In this case, the mutational landscape resembles a relatively low peak with a relatively flat surface surrounded by smooth slopes. Indeed, selection may favor populations that occupy lower peaks surrounded by less precipitous mutational chasms [40]. Consistently with this description, out of the thirty-one CTX-M-15 (CTX-M-3+D240G) derivative mutants, twenty-three had an increased fitness and five corresponded to CTX-M enzymes already found in nature (Figure 5, suppl. Table S1).

The P167 pathway assures a good adaptation (high fitness) to high concentrations of ceftazidime (MICs of 3->256 µg/ml *versus* 1.5–24 µg/ml for the D240G pathway), but it is *fragile* in yielding resistant variants able to survive in the presence of ceftazidime and cefotaxime. On the contrary, the D240 pathway offers lower levels of resistance to ceftazidime, but it is *robust* in yielding variants able to survive in the presence of both drugs. Therefore, more CTX-M-1 variants will probably emerge in the D240 genetic landscape. In addition, cells producing CTX-M variants carrying both P167S and D240G changes in all possible genetic backgrounds consistently showed lower MIC values to cefotaxime and/or ceftazidime than the correspondent P167S or D240G immediate ancestors (suppl. Table S1). These data support the hypothesis that P167 and D240 pathways represent independent routes of diversification [23].

A third possible diversification pathway is proposed, where neither P167S nor D240G were selected in the first stage of the process, and where neutral drift might be significant in the initial steps. The role of drift in early stages of β-lactamase evolution might be significant in conditions of low genetic load and high mutation rate in ancestral bacterial populations [41],[42]. Indeed, increased mutation rates have been frequently observed in strains harboring CTX-M β-lactamases [43],[44]. Although this path shows a very flat fitness landscape (next to neutrality) it allows us to explain the selection of several variants like CTX-M-36 and CTX-M-61 (Figures 6 and 7). Moreover, this pathway provides alternative routes for the selection of some CTX-M enzymes, such as CTX-M-23 or CTX-M-28 predicted in the P167 or D240 pathways respectively, suggesting that the interconnection between this route and the two previous trajectories would facilitate the emergence of new variants, as illustrated in Figure 8.

This study shows a high congruence between phylogenetic reconstruction analysis and step-by-step site-specific mutagenesis, allowing different presumptive evolutionary trajectories to be identified. Such combination of theoretical phylogenetic reconstruction and directed mutagenesis might be useful to analyze and eventually predict evolutionary antimicrobial resistance trajectories. Whereas the phylogenetic reconstruction analyses inform about the most likely trajectories actually followed based only on existing nucleotide changes, mutants experimentally constructed by site-directed mutagenesis were ordered according to their increased resistance phenotypes to ceftazidime and/or cefotaxime. This allowed the identification of a higher number of possible trajectories than those derived from the phylogenetic analysis, as it included all possible intermediate CTX-M alleles, though only some have actually been detected in nature. Therefore, we cannot rule out the existence of non-described variants belonging to other trajectories or the contribution of non-identified selective constraints. In addition, it should be noted that the fixation of a given intermediate variant is not necessarily required for the appearance and increase in frequency of a further fitness-increasing mutant. On the other hand, the successful distribution of a given CTX-M variant (increase in frequency) is influenced not only by its relative fitness, but also by the genetic platforms responsible for its mobilization (IS*Ecp1*, IS*CR1*, IS*26* and phage-like) and dissemination (integrons, transposons, plasmids and/or clones). This is the case of CTX-M-1, CTX-M-3, CTX-M-15 or CTX-M-32 enzymes, which are successfully disseminated in specific genetic units even though they do not constitute the most successful variants in our experimental conditions [15].

**Figure 9:**
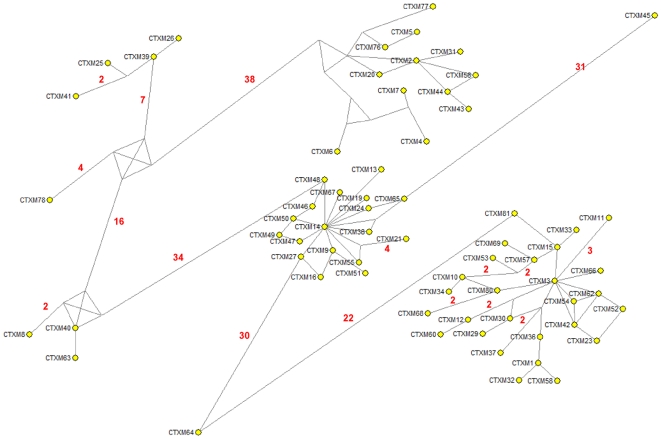
Median-joining network inferred for all CTX-M amino acid sequences studied. Numbers next to the segments connecting nodes denote the number of amino acid positions inferred to have changed between the two connected alleles, when these are larger than one. The major groups identified with this analysis correspond to the groups also defined in the phylogenetic tree shown in Figure 1.

The results shown in this work might well apply to other CTX-M clusters. An alternative reconstruction of the phylogenetic relationships among *bla*_CTX-M_ alleles was obtained by median-joining network analysis (Figure 9). This analysis revealed the central role of CTX-M-2 and CTX-M-14 in the diversification of the CTX-M-2 and CTX-M-9 clusters respectively, similar to that of CTX-M-3 in the CTX-M-1 cluster.

In conclusion, we suggest that the selective force pushing allelic diversification derived from CTX-M-3 was the ability of these CTX-M variants to provide better protection of the bacterial cells hosting random variant enzymes to the extended spectrum cephalosporins most frequently used in clinical settings [45]. Either in a synchronic or diachronic combination, both selective antibiotic drivers contribute to the increase in frequency of a wealth of mutational variants, so as to provide the necessary genetic background for a number of different accessible evolutionary paths. Antimicrobial agents should not be considered only as selectors for the most efficient resistance mechanisms but, by selecting at intermediate steps, they also act as diversifying agents of the evolutionary trajectories, since they contribute to shaping the adaptive landscape in which evolution has to occur.

## Materials and Methods

### CTX-M sequence alignments and phylogeny

Thirty-one *bla*_CTX-M-1_ group nucleotide sequences available at the GenBank database (http://www.ncbi.nlm.nih.gov) at the beginning of this study were analyzed. Sequences (n = 42) belonging to other CTX-M clusters (22 *bla*_CTX-M-9_, 12 *bla*_CTX-M-2_, 3 *bla*_CTX-M-8_ and 4 *bla*_CTX-M-25_, and the recombinant form *bla*_CTX-M-64_) along with 12 *bla*_KLU_ genes from putative *Kluyvera* spp. ancestors were included in order to perform a comprehensive analysis. The corresponding translated amino acid sequences were aligned using CLUSTALW [46], and gaps were inserted in the respective coding sequences in order to obtain a codon-based alignment which was used for the ensuing phylogenetic inference analyses. The alignment was manually revised and modified where necessary.

Bayesian and maximum likelihood phylogenetic trees were obtained using MrBayes 3.1.2 [47] and PHYML [48]. The Tamura-Nei nucleotide substitution model, selected with the ModelTest program [49] according to the Akaike information criterion (AIC), was used as evolutionary model and included a gamma distribution with six rate categories and a fraction of invariant sites to account for substitution rate heterogeneity among sites. The robustness of the maximum-likelihood topology was evaluated by the approximate likelihood ratio method [50] with 1000 bootstrap pseudorandom replicates. Additionally, a neighbor-joining tree [51] with 1000 bootstrap replicates was constructed using MEGA [52]. The topology resulting from likelihood analyses was used for the ensuing positive selection analyses after converting branch lengths to substitutions per codon-site.

An alternative method to reconstruct the genealogical relationships among closely related gene variants was also used. A median-joining network [53] was constructed using Networks version 4.5.1.0 (available at http://www.fluxus-technology.com) with amino acid sequences corresponding to all *bla*_CTX-M-1_ variants studied.

### Reconstruction of the CTX-M-1 cluster

In order to infer the most likely ancestral amino acid sequence at each internal node of the maximum-likelihood topology obtained, parsimony analyses of the 22 different positions among β-lactamases belonging to the CTX-M-1 cluster (12, 19, 23, 27, 35, 38, 67, 77, 89, 106, 114, 119, 140, 158, 167, 238, 240, 263, 271, 278, 279, 288, according to Ambler's numeration) [54] were performed using MESQUITE 2.6 [55]. We mapped these ancestral sequences onto the phylogeny and determined the extant sequences which retain the same states as the ancestral one.

### Models used to determine amino acid positions under positive selection in CTX-M-1 group enzymes

Amino acid positions in the CTX-M-1 cluster that were presumably subjected to positive selection were identified by the application of different selection tests using codon-based substitution models implemented in the PAML package [56]. According to these tests, the codon-by-codon ratio (ω) of the number of non-synonymous substitutions per non-synonymous site (dN) to the number of synonymous substitutions per synonymous site (dS), (ω = dN/dS) was calculated, with values of ω>1 indicating positive selection whilst values of ω<1 indicate purifying selection and values of ω = 1 represent neutral evolution.

The M7-M8 codon-based maximum-likelihood (ML) substitution models on an alignment of the CTX-M-3-like sequences were used in the analyses [57]. In the M7 model, the proportion of sites with different ω in the alignment is fitted by a β distribution with values between 0 (purifying selection) and 1 (neutral selection). The M8 model allows sites with ω>1, thus adding an extra class of sites, those evolving under positive selection, to the M7 model. A likelihood ratio test (LRT) was used in order to detect significantly better fit of the M8 model with respect to the M7, assuming a chi-squared null distribution. When the test was significant, Bayesian probabilities of the different site classes were calculated using a Bayes empirical Bayes procedure (BEB) [58] and those sites with an *a posteriori* probability of having a ω>1 higher than 0.95 were considered to have evolved under positive selection. Additionally, a branch-site model [59] was used to test whether the evolution of the CTX-M-1 cluster was accompanied by positive selection acting on some coding sites while the remaining clusters were under the constraint of ω≤1.

### Experimental validation of the phylogenetic reconstruction

According to the predictions endorsed by phylogenetic inference analyses, the phenotypic consequences of carrying one or several amino acid changes suspected to be under positive selection were investigated by site-directed mutagenesis on a *bla*_CTX-M-3a_ background. The analysis of each mutation alone or in combination with others allowed for experimentally testing the feasibility of the evolutionary trajectories followed to reach the most evolved variants according to the phylogenetic analysis (see below, evaluation of mutational trajectories). All predicted trajectories were explored by sequential site-directed mutagenesis [27]. A total of 47 variants were constructed in two steps by using primers A77V-F, N114D-F, A140S-F, P167S-F and D240G-F in combination with CTX-MPst-Rv, and primers A77V-R, N114D-R, A140S-R, P167S-R and D240G-R in combination with CTX-MEco-Fw (step 1), and primers CTX-MEco-Fw and CTX-MPst-Rv (step 2) to obtain each complete *bla*_CTX-M_ gene using fragments from the previous step as DNA template, as previously described [23]. The D288N change was obtained by conventional PCR, using CTX-MEco-Fw and D288N-R oligonucleotides. Oligonucleotide sequences used in this study are described in suppl. Table S2.

The different *bla*_CTX-M_ genes were cloned in pBGS18 (Kan^R^) plasmid vector [60] and used to transform *Escherichia coli* strain MI1443 (Δ*frdABCD*, Δ*ampC*, *recA*, *Sm^R^*) [61]. Briefly, amplified products of complete *bla*_CTX-M_ genes (step 2) and pBGS18 plasmid were purified (QIAquick purification and QIAprep Spin Miniprep kits, Qiagen, GmbH, Hilden, Germany), digested with enzymes *Eco*RI and *Pst*I, ligated and the recombinant plasmids transformed into MI1443 cells, using kanamycin (50 µg/ml) and cefotaxime (1 µg/ml) as selector agents. Plasmid DNA was extracted from one single clone per selective plate and re-transformed into MI1443 cells, selecting only with kanamycin (50 µg/ml), in order to avoid the selection of membrane permeability mutants. Recombinant plasmids were purified and the corresponding *bla* gene was sequenced to confirm the presence of the desired mutation [23].

### Antibiotic susceptibility testing

The MIC values for the different *E. coli* MI1443 strains carrying recombinant plasmids containing the different *bla*_CTX-M_ gene derivatives were determined by the E-test method, following manufacturer instructions (AB BioMérieux, Solna, Sweden). The antibiotics tested were: cefotaxime, ceftazidime, cefepime, cephalotin, cefuroxime, cefoxitin and amoxicillin-clavulanic acid (Sigma-Aldrich Inc., St. Louis, MO, EUA). The MIC of each transformant obtained and of the control strain MI1443 (pBGS18+*bla*_CTX-M-3_) were assayed in triplicate. The significance of the difference in MIC values between pairs of mutants was obtained with Mann-Whitney tests, since the number of replicates for each MIC estimate was too low for parametric testing. Evolutionary steps identified as significant according to this analysis are represented in Figures 4, 5 and 7.

### Evaluation of mutational trajectories

To compute the probability of each mutational trajectory, experimentally generated cefotaxime and ceftazidime MIC values were used as approximations to the fitness of each variant in the presence of the corresponding antibiotic. A probability proportional to the gain in fitness (increase in MIC value) was assigned to each step of each trajectory from the initial to the final variant for cefotaxime and ceftazidime. Under a selective environment, negative fitness values equaled zero, since selection cannot contribute to the fixation of a new, less fit variant. Estimates of the likelihood of each trajectory were obtained assuming the strong-selection weak-mutation (SSWM) model [62] according to Weinreich *et al.* [27]. Trajectories involving one or more steps with null probability of being favored by natural selection were considered to have zero chances of occurring under a selective framework.

### GenBank accession numbers

The nucleotide sequences of *bla*_CTX-M_ genes used for phylogenetic analysis were as follows: *bla*_CTX-M-1_, X92506; *bla*_CTX-M-2_, X92507; *bla*_CTX-M-3_, Y10278; *bla*_CTX-M-4_, Y14156; *bla*_CTX-M-5_, U95364; *bla*_CTX-M-6_, AJ005044; *bla*_CTX-M-7_, AJ005045; *bla*_CTX-M-8_, AF189721; *bla*_CTX-M-9_, AF174129; *bla*_CTX-M-10_, AF255298; *bla*_CTX-M-11_, AY005110; *bla*_CTX-M-12_, AF305837; *bla*_CTX-M-13_, AF252623; *bla*_CTX-M-14_, AF252622; *bla*_CTX-M-15_, AY044436; *bla*_CTX-M-16_, AY029068; *bla*_CTX-M-17_, AY033516; *bla*_CTX-M-18_, AF325133; *bla*_CTX-M-19_, AF325134; *bla*_CTX-M-20_, AJ416344; *bla*_CTX-M-21_, AJ416346; *bla*_CTX-M-22_, AY080894; *bla*_CTX-M-23_, AF488377; *bla*_CTX-M-24_, AY143430; *bla*_CTX-M-25_, AF518567; *bla*_CTX-M-26_, AY157676; *bla*_CTX-M-27_, AY156923; *bla*_CTX-M-28_, AJ549244; *bla*_CTX-M-29_, AY267213; *bla*_CTX-M-30_, AY292654; *bla*_CTX-M-31_, AJ567481; *bla*_CTX-M-32_, AJ557142; *bla*_CTX-M-33_, AY238472; *bla*_CTX-M-34_, AY515297; *bla*_CTX-M-35_, AB176534; *bla*_CTX-M-36_, AB177384; *bla*_CTX-M-37_, AY649755; *bla*_CTX-M-38_, AY822595; *bla*_CTX-M-39_, AY954516; *bla*_CTX-M-40_, AY750914; *bla*_CTX-M-41_, DQ023162; *bla*_CTX-M-42_, DQ061159; *bla*_CTX-M-43_, DQ102702; *bla*_CTX-M-44_, D37830; *bla*_CTX-M-45_, D89862; *bla*_CTX-M-46_, AY847147; *bla*_CTX-M-47_, AY847143; *bla*_CTX-M-48_, AY847144; *bla*_CTX-M-49_, AY847145; *bla*_CTX-M-50_, AY847146; *bla*_CTX-M-51_, DQ211987; *bla*_CTX-M-52_, DQ223685; *bla*_CTX-M-53_, DQ268764; *bla*_CTX-M-54_, DQ303459; *bla*_CTX-M-55_, DQ343292; *bla*_CTX-M-56_, EF374097; *bla*_CTX-M-57_, DQ810789; *bla*_CTX-M-58_, EF210159; *bla*_CTX-M-59_, DQ408762; *bla*_CTX-M-60_, AM411407; *bla*_CTX-M-61_, EF219142; *bla*_CTX-M-62_, EF219134; *bla*_CTX-M-63_, AB205197; *bla*_CTX-M-64_, AB284167; *bla*_CTX-M-65_, EF418608; *bla*_CTX-M-66_, EF576988; *bla*_CTX-M-67_, EF581888; *bla*_CTX-M-68_, EU177100; *bla*_CTX-M-69_, EU402393; *bla*_CTX-M-79_, EF426798; *bla*_CTX-M-80_, EU202673; *bla*_CTX-M-81_, EU136031; *bla*_CTX-M-82_, DQ256091; *bla*_KLUC-1_, AY026417; *bla*_KLUC-2_, EF057432; *bla*_KLUG-1_, AF501233; *bla*_KLUA-1_, AJ272538; *bla*_KLUA-2_, AJ251722; *bla*_KLUA-3_, AJ427461; *bla*_KLUA-8_, AJ427465; *bla*_KLUA-10_, AJ427467; *bla*_KLUA-12_, AJ427469; *bla*_KLUY-1_, AY623932; *bla*_KLUY-2_, AY623935; *bla*_KLUY-3_, AY824948.

## Supporting Information

Table S1 Comparison of P167S-D240G double mutants with their corresponding single mutant ancestors in all genetic backgrounds.
(0.09 MB PDF)

Table S2 Oligonucleotides used in this study.
(0.02 MB PDF)
