# Supplementary material for: Evolutionary Trajectories of Beta-Lactamase CTX-M-1 Cluster Enzymes: Predicting Antibiotic Resistance
Source: PLoS Pathog. 2010 Jan 22;6(1):e1000735. doi: 10.1371/journal.ppat.1000735 (PMC2809773; doi:10.1371/journal.ppat.1000735)
Supplement: Table S1 — Comparison of P167S-D240G double mutants with their corresponding single mutant ancestors in all genetic backgrounds. (0.09 MB PDF) [file ppat.1000735.s001.pdf]

Suppl. Table S1. Comparison of P167S-D240G double mutants with their corresponding single mutant ancestors in all genetic backgrounds.

		Amino acid changes [a]						MIC values (μg/ml) [b]	
**Mutant [c]**	**GenBank Acc. Nº [d]**	**A77V**	**N114D**	**A140S**	**P167S**	**D240G**	**D288N**	**CTX**	**CAZ**
000100	-				+			1	32
000010	-					+		128	2
000110	GU125678				+	+		1	2
100100		+			+			4	32
100010		+				+		>256	6
100110	GU125685	+			+	+		4	24
010100			+		+			8	>256
010010			+			+		256	12
010110	GU125688		+		+	+		0.5	3
001100				+	+			1	16
001010				+		+		64	8
001110	GU125681			+	+	+		0.75	1
000101					+		+	0.5	8
000011						+	+	128	4
000111	GU125691				+	+	+	0.5	2
110100		+	+		+			8	64
110010		+	+			+		>256	8
110110	GU125695	+	+		+	+		4	12
101100		+		+	+			4	48
101010		+		+		+		256	8
101110	GU125714	+		+	+	+		0.5	2

Suppl. Table S1. Cont.

		Amino acid changes [a]						MIC values (μg/ml) [b]	
**Mutant [c]**	**GenBank Acc. Nº [d]**	**A77V**	**N114D**	**A140S**	**P167S**	**D240G**	**D288N**	**Cefotaxime**	**Ceftazidime**
100101		+			+		+	2	48
100011		+				+	+	256	6
100111	GU125694	+			+	+	+	3	12
011100			+	+	+			0.5	12
011010			+	+		+		256	12
011110	GU125708		+	+	+	+		0.2	3
010101			+		+		+	1	16
010011			+			+	+	12	4
010111	GU125705		+		+	+	+	0.75	4
001101				+	+		+	2	32
001011				+		+	+	6	2
001111	GU125656			+	+	+	+	0.5	2
111100		+	+	+	+			3	96
111010		+	+	+		+		>256	12
111110	GU125709	+	+	+	+	+		3	8
110101		+	+		+		+	2	96
110011		+	+			+	+	>256	24
110111	GU125700	+	+		+	+	+	2	12
101101		+		+	+		+	1.5	32
101011		+		+		+	+	256	4
101111	GU125710	+		+	+	+	+	0.75	3

Suppl. Table S1. Cont.

		Amino acid changes [a]						MIC values (μg/ml) [b]	
**Mutant [c]**	**GenBank Acc. N° [d]**	**A77V**	**N114D**	**A140S**	**P167S**	**D240G**	**D288N**	**Cefotaxime**	**Ceftazidime**
011101			+	+	+		+	0.2	3
011011			+	+		+	+	12	1.5
011111	GU125711		+	+	+	+	+	0.2	0.75
111101		+	+	+	+		+	12	256
111011		+	+	+		+	+	256	8
111111	GU132745	+	+	+	+	+	+	3	12

[a] Amino acid changes corresponding to non-synonymous substitutions introduced by site-directed mutagenesis, "+" indicates presence. [b] Minimum Inhibitory Concentration (MIC) values as determined by E-test to the following β-lactam antibiotics: CTX, cefotaxime; CAZ, ceftazidime. [c] The binary code indicates the content of mutations in each CTX-M variant in this order: A77V, N114D, A140S, P167S, D240G and D288N. [d] GenBank accession numbers of *bla*$_{CTX-M}$ alleles corresponding to the double P167S-D240G mutants constructed by site-directed mutagenesis.
