# Supplementary material for: Evolutionary Trajectories of Beta-Lactamase CTX-M-1 Cluster Enzymes: Predicting Antibiotic Resistance
Source: PLoS Pathog. 2010 Jan 22;6(1):e1000735. doi: 10.1371/journal.ppat.1000735 (PMC2809773; doi:10.1371/journal.ppat.1000735)
Supplement: Table S2 — Oligonucleotides used in this study. (0.02 MB PDF) [file ppat.1000735.s002.pdf]

Suppl. Table S2. Oligonucleotides used in this study.

Oligonucleotides [a]	Sequence (5’-3’)	Size of amplification product (bp)	Reference
A77V-F	TGATGGCCGTGGCCGCGGTGCTGAA	654	[23]
A77V-R	TTCAGCACCGCGGCCACGGCCATCA	300	[23]
N114D-F	AAGCACGTCGATGGGACGATG	544	This study
N114D-R	TGACATCGTCCCATCGACGTG	409	This study
A140S-F	ATGAATAAGCTGATTTCTCACG	472	This study
A140S-R	AACGTGAGAAATCAGCTTATTC	481	This study
P167S-F	CGTACCGAGTCGACGTTAA	385	[23]
P167S-R	TGTTTAACGTCGACTCGGTA	566	[23]
D240G-F	AAACCGGCAGCGGTGGCTAT	174	[23]
D240G-R	ATAGCCACCGCTGCCGGTTT	776	[23]
CTX-M-Eco	GGAATTCGACTATTCATGTTGTTGTTAATT [b]	-	[23]
CTX-M-Pst	AACTGCAGTTCCGCTATTACAAACCGT	-	This study
D288N	AACTGCAGATTACAAACCGTTGGTGACGAT	923	This study

[a] These primers were designed based on *bla*$_{\text{CTX-M-1}}$ gene sequence (GenBank accession number X92506); [b] The *Eco*RI and *Pst*I restriction sites are underlined in primers CTX-M-Eco, CTX-M-Pst and D288N. PCR products were obtained using CTX-M-Eco and CTX-M-Pst or D288N with reverse and forward primers, respectively.
